# Surgery

**Published:** 1896-09

**Authors:** John Parmenter

**Affiliations:** Professor of anatomy and clinical surgery, University of Buffalo


### SURGERY.

CONDUCTED BY JOHN PARMENTER, M. D.,
Professor of anatomy and clinical surgery, University of Buffalo.

### SPRAINED ANKLES.

CROOK, J. L., (*International Journal of Surgery*, July, 1896,) contributes an interesting but brief article upon the treatment of sprained ankles. After touching briefly upon the pathology and symptomatology of this condition, he describes his method of dealing with it. The procedure employed is a modification of that previously advocated by Gibney, who claimed for it that it "involves no loss of time, requires no crutches, and is not attended with any ultimate impairment of functions." Crook's method, given in his own words, is as follows :

A number of strips of rubber adhesive plaster about nine to twelve inches in length and of appropriate width are prepared. Beginning at the outer border of the foot, near the little toe, the first strip partially encircles the joint and ends behind the foot. The second strip is begun on the inner side of the foot and is applied on the opposite side, nearly meeting the first strip behind. Other strips are applied in like manner, each one overlapping the last, and crossing its fellow of the opposite side in front, so that the ankle is snugly and smoothly encased, care being taken not to completely encircle the joint with any one strip. After having bound the foot firmly, it is well to add one broad strip running around the foot, from the internal side of the leg down the internal side of the foot, across the plantar surface and up the outside of the leg, "as much as possible to take the place of the middle fasciculus of the external lateral ligament which is so often the one most injured." It is a good plan to place a pad of absorbent cotton over the external malleolus and in the fossa below, to prevent undue pressure and chafing. Any one of the injured ligaments may receive a similar reinforcement from an extra strip. I then apply a roller smoothly over the entire surface, allowing it to remain until the plaster takes firm hold.

The writer then cites several cases illustrating the value of his method. His favorable experience leads him to extol this treatment, a feeling in which all surgeons who have given the procedure a trial will concur.

---

### PYLEPHLEBITIS SUPPURATIVA.

MYNTER (*International Journal of Surgery*, April, 1896,) describes a case of pylephlebitis suppurativa occurring in a patient suffering from appendicitis. Operation upon the fifth day revealed an appendix seven inches long, totally gangrenous and perforated at its tip, which lay in the pelvis. Death occurred on the fourth day following operation, the symptoms on the last day being as follows : Temperature, 101° ; pulse, 92 ; abdomen soft, but tender to pressure upward ; no symptoms of peritonitis ; no swelling of liver or spleen ; had had a large black stool. Icterus was marked, urine scanty and full of bile pigment. Patient became semi-comatose with stertorous respiration and vomited continuously, in spite of irrigation, a dark fluid, and finally died in coma. No post mortem was permitted, but the diagnosis seems to the writer to be fully justified by the history and symptoms. He regards the disease as generally secondary to suppurating processes in the abdomen, the portal vein becoming infected, either through the formation of septic thrombi in its radicles, or from emboli in the larger branches, producing retrograde thrombosis, followed by phlebitis. Appendicitis is the most frequent cause through infection of a mesenteric vein when the mesentery or omentum is adherent to the gangrenous appendix. Other causes, however, may be found in gastric and intestinal ulcers, phlebitis of the hemorrhoidal veins, dysentery, abscess of the spleen, suppurating mesenteric glands, or localised peritonitis around the hepatico-duodenalis ligament. The liver itself may be the starting point of the disease. The symptoms of pylephlebitis are fairly well pronounced and depend less upon occlusion of the portal system than upon septic and thrombo-pyemic, preceded by the symptoms of the original disease which caused the phlebitis. They include pain, violent chill, painful enlargement of the liver and spleen, icterus, bloody vomiting and peritonitis, and the more in number these symptoms are present the more probable is the diagnosis. The malady is uniformly fatal and, therefore, treatment is unavailing.

---

### CONSERVATIVE OPERATIVE TREATMENT OF SACCULATED KIDNEY—CYSTONEPHROSIS.

Fenger, (*Annals of Surgery*, June, 1896,) in a masterly article, makes a strong plea for more conservatism in dealing with this form of kidney lesion. After considering the mechanical and pathological aspects of cystonephrosis, he describes an illustrative case and, as a result of his study of, and experience with, this condition, he reaches the following conclusions :

Conservative surgery in cystonephrosis demands a number of successive operations.

I. *Hydronephrosis.*—(1) Lumbar nephrotomy, followed by packing and aseptic drainage. If urinary fistula remains after three months.

(2) Operation for the stenosis—namely, for (*a*) stricture of the ureter, or (*b*) valve-formation and oblique insertion. If the fistula still remains with the ureter patent, which occurs only when there is obstruction above the ureter.

(3) Operation for sacculated kidney as designed by me—namely, bisection of the kidney and division of the partition walls between sacs. When the entire territory of the sac is thus laid open and the ureter is patent, as demonstrated by free passage of bougies from the kidney to the bladder, and by free passage of injected fluid, then

(4) Closure of the fistula by reunion of the bisected kidney. This last operation may confidently be expected to be followed by disappearance of the fistula. It should not, however, be done until the pyelitis, if present, has been cured by thorough irrigation from the kidney to the bladder. Küster observed a case of nephrotomy in which the fistula closed spontaneously, with patent ureter, but the pyelitis persisted, giving an incomplete cure. I have seen, however, that when we closed the fistula before the pus has disappeared entirely from the urine, the pus in the urine having remained unchanged in amount for a considerable time, that the closure of the fistula acts as a curative measure and causes the pyelitis to cease. Is it by exclusion of air or by cessation of irritation of the drainage-tube, the cause of the pyelitis having been removed, as shown in my two cases reported in a former paper?

In unilocular hydronephrosis the operation for bisection of the sacculated kidney is, of course, not required, and only nephrotomy, the operation for valve-formation in the ureter, and that for closure of the kidney, are needed.

II. *Pyonephrosis.*—(1) Lumbar nephrotomy, followed by drainage and local treatment of the pyelonephritis. When the fistula persists, when considerable healthy urine passes through it and the kidney has retracted to a reasonable degree,—that is, in two or three months.

(2) Search for the obstruction, and operation to remove it. (*a*) Stricture of the ureter and valve-formation at its pelvic entrance. If this condition, with a ureter patent from pelvis to bladder, still does not prevent the passage of large quantities of urine through the lumbar fistula, obstruction above the pelvis should be suspected, and (*b*) operation for sacculated kidney, with bisection and division of the partition walls of the calyces and of the branches of the ureter. When the whole territory of the secreting kidney tissue is thus an unimpeded connection with the bladder, and still spontaneous closure does not take place, although the pyelitis is almost cured, the final step to effect closure of the fistula should be taken—namely,

(3) Closure of the bisected kidney, by loosening the adhesion of its two halves to the abdominal wall and by suturing the kidney substance together.

Nephrectomy, like amputation, is a short and summary way to deal with the diseased organ or limb. The loss of a kidney, even if half its function remain, may be more dangerous to life than the loss of an arm or a leg. At the present day we would not think of sacrificing an extremity which might be of even partial use, but kidneys have been sacrificed by the hundreds, as is shown by the number of cases of primary nephrectomy in cystonephrosis (pyo- or hydronephrosis). It is impossible to estimate how many of these kidneys might have been saved by conservative operation.

Conservative operation, to save a cystonephrotic kidney, requires a hard struggle, many operations during a long period of time, and a great deal of energy and patience on the part of both patient and surgeon; but the case above detailed demonstrates that when this course of treatment has to be carried out, because nephrectomy would mean death, a final success can be obtained and the patient be saved from the dangers of, and the social misery attendant upon, an abundantly secreting urinary fistula.

